# High Order Gene-Gene Interactions in Eight Single Nucleotide Polymorphisms of Renin-Angiotensin System Genes for Hypertension Association Study

**DOI:** 10.1155/2015/454091

**Published:** 2015-04-19

**Authors:** Cheng-Hong Yang, Yu-Da Lin, Shyh-Jong Wu, Li-Yeh Chuang, Hsueh-Wei Chang

**Affiliations:** ^1^Department of Electronic Engineering, National Kaohsiung University of Applied Sciences, Kaohsiung 80778, Taiwan; ^2^Department of Medical Laboratory Science and Biotechnology, Kaohsiung Medical University, Kaohsiung 80708, Taiwan; ^3^Department of Chemical Engineering & Institute of Biotechnology and Chemical Engineering, I-Shou University, Kaohsiung 84001, Taiwan; ^4^Cancer Center, Translational Research Center, Kaohsiung Medical University Hospital, Kaohsiung Medical University, Kaohsiung 80708, Taiwan; ^5^Institute of Medical Science and Technology, National Sun Yat-sen University, Kaohsiung 80424, Taiwan; ^6^Research Center of Environmental Medicine, Kaohsiung Medical University, Kaohsiung 80708, Taiwan; ^7^Department of Biomedical Science and Environmental Biology, Kaohsiung Medical University, Kaohsiung 80708, Taiwan

## Abstract

Several single nucleotide polymorphisms (SNPs) of renin-angiotensin system (RAS) genes are associated with hypertension (HT) but most of them are focusing on single locus effects. Here, we introduce an unbalanced function based on multifactor dimensionality reduction (MDR) for multiloci genotypes to detect high order gene-gene (SNP-SNP) interaction in unbalanced cases and controls of HT data. Eight SNPs of three RAS genes (angiotensinogen, *AGT*; angiotensin-converting enzyme, *ACE*; angiotensin II type 1 receptor, *AT*_1_ *R*) in HT and non-HT subjects were included that showed no significant genotype differences. In 2- to 6-locus models of the SNP-SNP interaction, the SNPs of *AGT* and *ACE* genes were associated with hypertension (bootstrapping odds ratio [Boot-OR] = 1.972~3.785; 95%, confidence interval (CI) 1.26~6.21; *P* < 0.005). In 7- and 8-locus model, SNP A1166C of *AT*_1_ *R* gene is joined to improve the maximum Boot-OR values of 4.050 to 4.483; CI = 2.49 to 7.29; *P* < 1.63*E* − 08. In conclusion, the epistasis networks are identified by eight SNP-SNP interaction models. *AGT*, *ACE*, and *AT*_1_ *R* genes have overall effects with susceptibility to hypertension, where the SNPs of *ACE* have a mainly hypertension-associated effect and show an interacting effect to SNPs of *AGT* and *AT*_1_ *R* genes.

## 1. Introduction

The renin-angiotensin system (RAS) represents a critical endocrine regulator for maintaining blood pressure and blood fluid volume in the circulatory system. Single nucleotide polymorphisms (SNPs) of* RAS* genes such as angiotensinogen (*AGT*) [1, 2], angiotensin-converting enzyme (*ACE*) [3, 4], and angiotensin II type 1 receptor (*AT*_1_ *R*) [5, 6] are known to be associated with cardiovascular diseases [7–9]. For example, the SNP G-217A of* AGT* gene but not the SNPs A-6G and M235T of* AGT* gene may associate with hypertension in patients from Taiwan [1]. The I allele of* ACE* gene and +1166 C allele of* AT*_1_ *R* gene are reportedly associated with hypertension [10]. However, these studies were mainly relying on the association with hypertension using single SNP models and rare SNP effects were commonly ignored.

Accumulating evidence indicates that high order gene-gene (SNP-SNP) interaction can deeply affect disease susceptibility. For example, the A1166C of* AT*_1_ *R* gene and I/D of* ACE* gene have synergistic effects on acute myocardial infarction [11]. The interactions between T174M, M235T, G-6A, A-20C, G-152A, G-217A of* AGT* gene, I/D of* ACE* gene, and A1166C of* AT*_1_ *R* gene have been examined in coronary artery disease [12]. A significant effect of gene-gene interaction in coronary artery disease was detected for G-217A and M235T of* AGT* gene and I/D of* ACE* gene. Additionally, joint effects of gene-gene interactions were discovered in blood pressure regulation [13], left ventricular mass [14], and acute myocardial infarction [11]. However, detecting gene-gene interactions remains a challenge due to a large number of possible SNP combinations.

To date, several computational methodologies have been proposed to detect the epistasis in many association studies [15–22]. Data mining and statistical analysis are a common approach to overcome computational challenges in detecting complex gene-gene interactions. For example, multifactor dimensionality reduction (MDR), a nonparametric statistical method, is commonly used for detecting possible gene-gene interactions in multigene causing diseases [23, 24]. However, this common MDR is only suitable for a balanced number of cases and controls. The original data sets of many association studies are usually unbalanced. Therefore, some information in real data set might get lost after resampling.

Here, we describe a case-control study of hypertension susceptibility that specifically evaluates gene-gene interactions using unbalanced function based MDR [25] that combines traditional statistical methods with novel computational algorithms. The unbalanced function based MDR uses the ratio between the percentages of cases in each genotype combination of case data and the percentage of controls in each genotype combination of control data. This is to classify by MDR classifier, to analyze possible gene-gene interactions associated with hypertension. Subsequently, the misclassification errors of multiple SNPs associated with high or low risks of hypertension can be computed. To examine the high order SNP-SNP interactions of RAS genes in hypertension, 8 SNPs were chosen, namely, the T174M/M235T/G-6A/A-20C/G-152A/G-217A of* AGT* gene, I/D of* ACE* gene, and A1166C of* AT*_1_ *R* gene. We aimed to find out the influence of these 8 SNPs on hypertension outcomes. The results show that unbalanced function based MDR can avoid the drawback of common MDR in an unbalanced real data set. Thus, the best unbalanced function based MDR model can correctly predict high order SNP-SNP interactions of hypertension susceptibility using real data sets.

## 2. Methods

The MDR method was briefly introduced and the unbalanced function based MDR method was explained in detail as follows.

### 2.1. MDR

**Figure 1:**
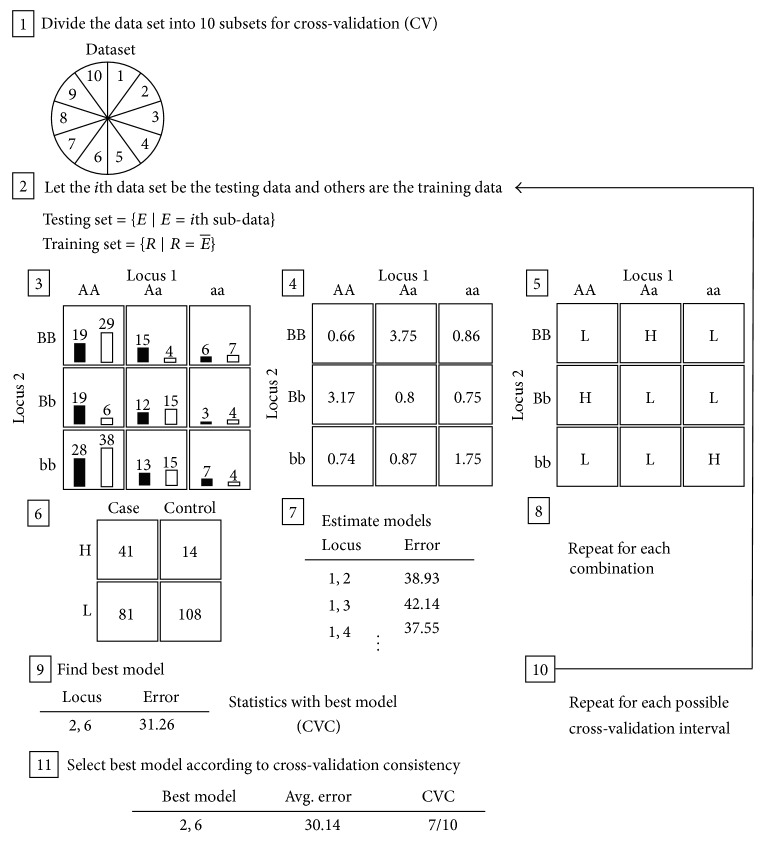
MDR flowchart. Eleven steps are described in Section 2.

In 2001, Ritchie et al. proposed a MDR to detect the potential gene-gene interaction. MDR is a robust nonparametric method that detects nonlinear interactions among multiple discrete genetic factors [23]. It is accomplished by data classifier technology to combine two or more attributes into a single attribute. Thus, representation of data space can be changed, and high-order gene-gene interactions can be evaluated by statistical classifiers.  Figure 1 illustrates the MDR procedure that produces the best model by the following algorithm.

**Step 1 .**

Divide the data set into 10 subsets for cross-validation (CV).

**Step 2 .**

Keep the *i*th data set as the testing data and others are the training data.

**Step 3 .**

Calculate the total number of cases and the total number of controls within each multifactor class.

**Step 4 .**

Evaluate the ratio between cases and controls in each genotype combination (i.e., a cell in *n* × *n* grid).

**Step 5 .**

Determine the ratio of high (H)/low (L) risks in each multifactor class. If the cases/controls ratio particular threshold, it is labeled with “H”; otherwise it is labeled with “L”.

**Step 6 .**

Compute the four frequencies of true positive (TP), false positive (FP), true negative (TN), and false negative (FN) in a 2-way contingency table.

**Step 7 .**

Evaluate the misclassification error.

**Step 8 .**

Repeat for each combination.

**Step 9 .**

Select the best model according to minimum misclassification error and record it into cross-validation consistency (CVC).

**Step 10 .**

Repeat for each CV interval.

**Step 11 .**

Select the best model according to the model with the highest frequency in CVC.

In MDR procedure, the original data are randomly sorted and divided into 10 subsets for CV. In each CV interval, 9 of 10 subsets are classified as the training data and the remaining one as independent testing data. The *n* loci and a possible multiloci class are represented in the following *n*-dimensional space:

(1) $$\begin{array}{c} L=\left\{l_{1},l_{2},l_{3},\ldots ,l_{n}\right\}. \end{array}$$

The value of *n* is designated depending on the number of factors being considered. Then, a set of *n* genetic factors is selected. The total numbers of cases or controls are counted in the multifactor class, and the ratio of the numbers of cases to controls is calculated. From (2), the multifactor class count and ratio can be obtained as follows:

(2) $$\begin{array}{c} f\left(L\right)=\frac{\sum_{j=1}^{P^{*}}u\left(L,P_{j}\right)}{\sum_{j=1}^{N^{*}}u\left(L,N_{j}\right)}, \end{array}$$

where

(3) $$\begin{array}{c} u\left(L,A\right)=\left\{\begin{array}{cc} 1 & \forall l\in A,\\ 0 & \forall l\notin A, \end{array}\right.\;\forall l\in L, \end{array}$$

where acronyms represent the following: *P*: the case data set; *N*: the control data set; *P* ^∗^: the number of case group in the training set; *N* ^∗^: the number of control group in the training set; *L*: the vector of variable combinations.

The function *u*( ) indicates a match if all parameters *l* in vector *L* match their cases or controls, then given a score of “1,” otherwise, given the score “0.”

Next, the high/low risk in each multifactor class is determined. Each multifactor class in *n*-dimensional space is labelled as “H” or “L” symbol. Label “H” indicates that ratio in the multifactor class meets or exceeds a particular threshold (high-risk group); otherwise, label is “L” (low-risk group). The threshold is equal to the one in a balanced data set. Thus, the huge genotype combinations in *n*-loci are reduced into a 2-way contingency table (TP, FP, TN, and FN) and allow the statistical analysis to evaluate the *n*-loci effect. MDR uses the misclassification error to evaluate a model value where the misclassification error function is calculated as follows:

(4) $$\begin{array}{c} f\left(C\right)=\frac{\text{FN}+\text{FP}}{\text{TP}+\text{FN}+\text{FP}+\text{TN}}, \end{array}$$

where acronyms represent the following: TP: the total number of labeled “H” in the case data; FP: the total number of labeled “H” in the control data; FN: the total number of labeled “L” in the case data; TN: the total number of labeled “L” in the control data.

After all the multifactor combinations are evaluated by misclassification error, the MDR model with the minimum error rate individual is chosen and the model is considered the best model of training data at *i*-fold. The training model is then used to test the testing data and record the TP, FP, FN, and TN for evaluating the statistical power. Thus, MDR repeats the above procedure in each CV interval. The MDR model with the lowest number of misclassified individuals is chosen and ten best models are classified by the same combination in the CVC. Finally, the highest occurring frequency in CVC is considered the best model. If a tie between 2 or more models happens, then the first appearing model is considered the best model.

### 2.2. Unbalanced Function Based MDR

In unbalanced function based MDR [25], the ratio between the percentages of cases in each genotype combination of case data (i.e., a cell in *n* × *n* grid for cases) to the percentages of controls in each genotype combination of control data (i.e., a cell in *n* × *n* grid for controls) is proposed to classify the data to the high- and low-risk groups. Thus, the highest ratio between case and control groups can be clearly detected. The strategy is to modify the ratio between cases and controls in the ratio function of MDR, that is, (2). The following equation is introduced to calculate the ratio (percentage) of cases to controls:

(5) $$\begin{array}{c} f\left(L\right)=\frac{N^{*}\left[\sum_{j=1}^{P^{*}}u\left(L,P_{j}\right)\right]}{P^{*}[\sum_{j=1}^{N^{*}}u\left(L,N_{j}\right)]}, \end{array}$$

where

(6) $$\begin{array}{c} u\left(L,A\right)=\left\{\begin{array}{cc} 1 & \forall l\in A,\\ 0 & \forall l\notin A, \end{array}\right.\;\forall l\in L, \end{array}$$

where acronyms represent the following: *P*: the cases data set; *N*: the control data set; *P* ^∗^: the number of case group in the training set; *N* ^∗^: the number of control group in the training set; *L*: a vector of variable combinations.

The function *u*( ) is a match (given a score of “1”) if all parameters *l* in vector *L* match their cases or controls; a mismatch is given the score “0.”

Our strategy is to modify the misclassification error rate function of MDR, that is, (4). Equation (7) proposed by Velez et al. [26] is introduced into MDR; therefore, the two classes are equally responsible for both positive and negative errors due to the class imbalance. The equation evaluates the misclassification error rate according to the arithmetic mean of sensitivity and specificity. The adjusted misclassification error is algebraically identical to the error rate if the data set is imbalanced. Consider

(7) $$\begin{array}{c} f\left(C\right)=0.5\times \left(\frac{\text{FN}}{\text{TP}+\text{FN}}+\frac{\text{FP}}{\text{FP}+\text{TN}}\right), \end{array}$$

where acronyms represent the following: TP: the total number of labeled “H” in the case data; FP: the total number of labeled “H” in the control data; FN: the total number of labeled “L” in the case data; TN: the total number of labeled “L” in the control data.

Here we provide an example to show how the unbalanced function based MDR works (see Supplementary File in Supplementary Material available online at http://dx.doi.org/10.1155/2015/454091).

### 2.3. Study Population

This was a single center, case-control study. A detailed description of the subject collection has been published previously [1, 3]. In brief, hypertensive and normotensive patients (HT and non-HT subjects) were recruited from an outpatient clinic of the National Taiwan University Hospital from July 1995 through June 2002. The non-HT subjects were from the same areas as the hypertensives and had no history of hypertension, diabetes mellitus, renal insufficiency, significant hepatic disease, or apparent coronary artery disease. The basic characteristics of the HT and non-HT groups have been described previously [27] and are shown in Table 1. The demographic and laboratory data were collected from the medical chart records. The study protocols were reviewed and approved by a local institutional committee. All subjects gave informed consent as approved by the institutional review board at this hospital.

### 2.4. Statistical Analysis

The power statistical analysis was implemented by the G^∗^ power 3.1.5 tool [28, 29]. The SNPs were evaluated by their odds ratios (OR), 95% CI, and *P* values. OR was used to measure the risk of disease; *P* values indicate significant differences between the cases and controls. All statistical analyses were implemented using SPSS version 19.0 (SPSS Inc., Chicago, IL).

## 3. Results

### 3.1. Data Set

The hypertension data set with hypertension (*n* = 313) and nonhypertension (*n* = 130) was obtained from our previous study [27]. The complete genotype data set is available at http://bioinfo.kmu.edu.tw/non-HT_and_HT_genotype_data.xlsx. Eight SNPs were included: T174M (rs4762), M235T (rs699), G-6A (rs5051), G-217A (rs5049), G-152A (rs11568020), A-20C (rs5050), I/D (rs4646994), and A1166C (rs5186) of three RAS genes (*AGT*,* ACE*, and* AT*_1_ *R*). However, the possible SNP-SNP interaction was not examined. Here, we used the unbalanced function based MDR with minimum misclassification error rate to identify the best SNP-SNP interaction model with significant differences between hypertension (HT, cases) and nonhypertension (non-HT, controls) groups.

Table 1 shows the basic characteristics of the HT and non-HT groups. HT patients had a significantly higher risk for male gender, age, systolic blood pressure (BP(S)), diastolic blood pressure (BP(D)), and cholesterol. Body height, body weight, body mass index, cigarette smoking, and triglyceride were similar between HT and non-HT groups. The age, systolic blood pressure, diastolic blood pressure, and cholesterol of the hypertensives were significantly higher than those of the normotensives in Table 1.

### 3.2. Single-Locus Analysis

Table 2 shows the performance (*P* values of chi-square test) of each individual SNP. Among these eight SNPs, most individual SNPs paired with any genotype show no significant difference (*P* > 0.05) between the HT and non-HT groups. The frequency difference of SNP I/D of* ACE* gene between HT and non-HT groups is significant when based on chi-square test (ID and DD, *P* = 0.031 and 0.010, resp.). However, it is not significant after a Bonferroni correction (*P* > 0.006, i.e., 0.05/8).

### 3.3. Multilocus Analyses: Determination of the Best Model

All significant 2-locus SNP-SNP interactions (Table 3) are known to define the epistasis risk score which are collectively referred to as an epistasis network. Although some 2-locus models have higher OR values and lower *P* values, the best model was selected according to the model with the highest frequency in CVC which consisted of the model with the lowest error rate in each CV. Among these models, the lowest error of the best model is 0.419 for a 2-locus model (*AGT* G-217A +* ACE* I/D). Similarly, all the best models in 3- to 8-locus models are listed in Table 4.

### 3.4. Multiloci Analysis: Error Rates

Table 4 summarizes the results of the unbalanced function based MDR analysis for the best 2- to 8-locus models. Consistency data indicate that including more SNPs leads to a higher occurrence of hypertension. As the loci number increases, the prediction error rates were reduced from 41.9 to 32.6. In other words, the correct prediction was 58.1~67.4%. An 8-locus model had a minimum prediction error of 32.6%. Based on the null hypothesis of no association, it is impossible that an error rate ≤32.6 is observed by chance in randomized data. The 2- to 6-locus models suggest that those SNPs of the* AGT* and* ACE* genes were associated with hypertension. Both 7-locus and 8-locus models suggest that the listed SNPs in* AGT*,* ACE*, and* AT*_1_ *R* genes were important in association with hypertension. Additionally, power analysis represents the degree of rejection for H0 that is significant at *α* = 0.05. Applying the MDR method, the testing data results were always not significant (at *P* > 0.05). Thus, we defined H0 as the result of the test set is the same as for the training set (H0), and H1 shows that the result of the test set was different from the training set. The powers in 2- to 8-locus, ranging from 0.901 to 0.999, indicate that occurrence probabilities in all models are higher than 0.9. These findings suggest that all these 8 SNPs are significantly associated with hypertension.

### 3.5. Multiloci Analysis: OR and Boot-OR

In Table 4, the occurrences of frequency differences between HT and non-HT groups are different, the best 2- to 8-locus models generated from unbalanced function based MDR are significant (*P* < 0.01, data not shown). The OR values in 2- to 8-locus models increase from 2.054 to 4.628. For the implementation of bootstrapping in 1000 samples, the adjusted OR (Boot-OR) values increase from 1.972 to 4.483 in 2- to 8-locus models and the *P* values of 2- to 8-locus models decrease from 0.003 to 1.48E-09. Both OR and Boot-OR values gradually increase when the loci numbers increase indicating that the hypertension risk is increasingly raised by the joint effect of SNPs. It also suggests the SNPs of* AGT*,* ACE*, and* AT*_1_ *R* genes are highly associated with hypertension risk. SNPs G-217A (*AGT*) and I/D (*ACE*) occur in all best models of 2 to 8 loci in association with hypertension. The associated effects of A1166C (*AT*_1_ *R*) are detected at the best 7- and 8-locus models and this leads to the highest risk compared to other models.

## 4. Discussion

Many important genes associated with hypertension were reported [30–32], but most of them are based on a single-SNP model and the potential joint effects of multiloci models were less addressed. The single-SNP model of our current study, I/D (*ACE*), is significantly associated with hypertension using the chi-square test. However, it is nonsignificant after Bonferroni's correction (Table 2). However, it was reported that nonsignificant SNPs when combined generate joint effects that are associated with diseases [33]. Thus, the effects of some SNPs may be ignored in a single-SNP model. Accordingly, gene-gene interaction analysis was chosen in this study to identify the possible joint effect of these nonsignificant SNPs in association with hypertension.

MDR is a robust analysis for a gene-gene interaction based on detecting nonlinear multigene interactions. MDR also limits the balanced study population to, respectively, determine the high and low risk for cases and controls. Therefore, the MDR is unsuitable for the majority of natural data sets which commonly belong to imbalanced cases and controls. The threshold *T* = 1 can effectively distinguish between high and low risks in each genotype combination (i.e., a cell in *n* × *n* grid of high- and low-risks) of MDR (steps 4 and 5 in Figure 1) but faults in the imbalanced data set. Although resampling techniques are widely applied to fit for MDR detecting epistasis in imbalanced data sets, the possible information missing by resampling is hard to be excluded.

**Figure 2:**
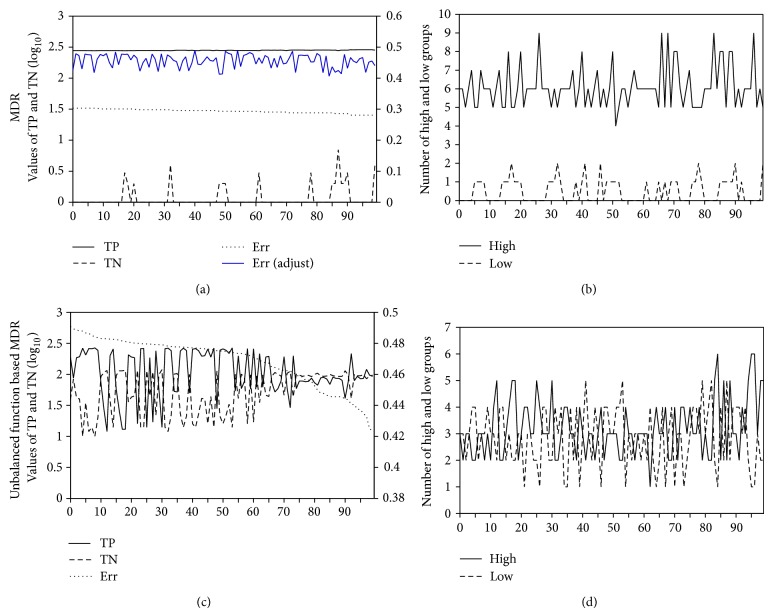
Comparison of the performance of MDR and the unbalanced function based MDR with the example of 2-locus SNPs. (a and c) Frequencies of TP, TN, and misclassification error. (b and d) The numbers of high- and low-risk groups. (a and c) The left scale of vertical axis is the log_10_ value for the total numbers of TP and TN. The right scale of vertical axis is the error rate. Blue line is the error rate based on (7), denoted by “Adjust Err”. The horizontal axis represents the 100 different models in 2-locus combinations, in which the models are sorted by error rate and selected by systematic sampling from all models. (b and d) The high/low lines indicate the distribution difference between the numbers of high- and low-risk groups.

In contrast, we demonstrated that our proposed unbalanced function based MDR is suitable for an imbalanced data set. For example, Figure 2 illustrates details of the computational process such as TP, TN, misclassification error rate, the total number of high-risk groups, and the total number of low-risk groups, which were obtained from a 2-locus SNP-SNP interaction model in MDR and unbalanced function based MDR. Figures 2(a) and 2(c) show the processes of the model selections and Figures 2(b) and 2(d) are the corresponding details for the models of Figures 2(a) and 2(c) in terms of the numbers of high- and low-risk groups. In Figure 2(a) for MDR, values of error rates show around 0.3 in 100 models of MDR, and TPs are always higher than TNs. Although TPs are slowly increased in all models, all error rates do not remain improved clearly. At the best model of MDR, the sensitivity and specificity are 0.0089 and 1, respectively. In Figure 2(c), for unbalanced function based MDR, the TNs are not always higher than the TPs. The error rates are clearly improved when there are small difference values between TP and TN. The sensitivity and specificity of the best model are 0.667 and 0.495, respectively.  Figure 2(b) for MDR clearly shows that high-risk groups in 100 models are always higher than low-risk groups due to the imbalanced data between cases (*n* = 313) and controls (*n* = 130) whereas Figure 2(d) for the unbalanced function based MDR shows the better frequencies of the numbers of high- and low-risk groups. Thus, (5) and (7) are effective in overcoming the MDR detecting multiloci interactions in imbalanced data sets. In summary, MDR may fail to correctly assign genotypes of multiloci to either high- or low-risk groups and does not provide correct error rates when the datasets are unbalanced. Thus, low error rate and high OR value of MDR may be due to its high TN. However, its TP value is low and indicates a low sensitivity for disease detection.

For hypertension association,* AGT* gene haplotype (T174M, M235T, G-6A, A-20C, G-152A, and G-217A) had been reported to interact with I/D of the* ACE* gene [3]. However, the role of A1166C of* AT*_1_ *R* gene in interacting with this* AGT* gene haplotype was not investigated. Because the six SNPs in the* AGT* gene are bound together due to its haplotype environment their potential interaction to SNPs of other genes may be limited. However, the joint effect of multiple SNPs between different genes may have a higher association degree than that of a single significant SNP. In the example of a natural data set with an unbalanced HT group and non-HT group, the unbalanced function based MDR algorithm is able to detect the significant association with hypertension in terms of 2- to 8-locus models by their OR and Boot-OR values (Table 4). The hypertension associated performance of all SNPs is additive from 2- to 8-locus models. The SNP appearing order is the same as the order of SNP joint effects in terms of the additive OR value (i.e., OR_*n*+1_ − OR_*n*_; Table 4) as follows: SNPs* ACE* I/D = G-217A > G-6A (2.372 − 2.054 = 0.318) > T174M (2.810 − 2.372 = 0.438) > G-152A (3.241 − 2.810 = 0.431) > A-20C (3.863 − 3.241 = 0.622) >* AT*_1_ *R* A1166C (4.510 − 3.863 = 0.647) > M235T (4.628 − 4.510 = 0.118).

**Figure 3:**
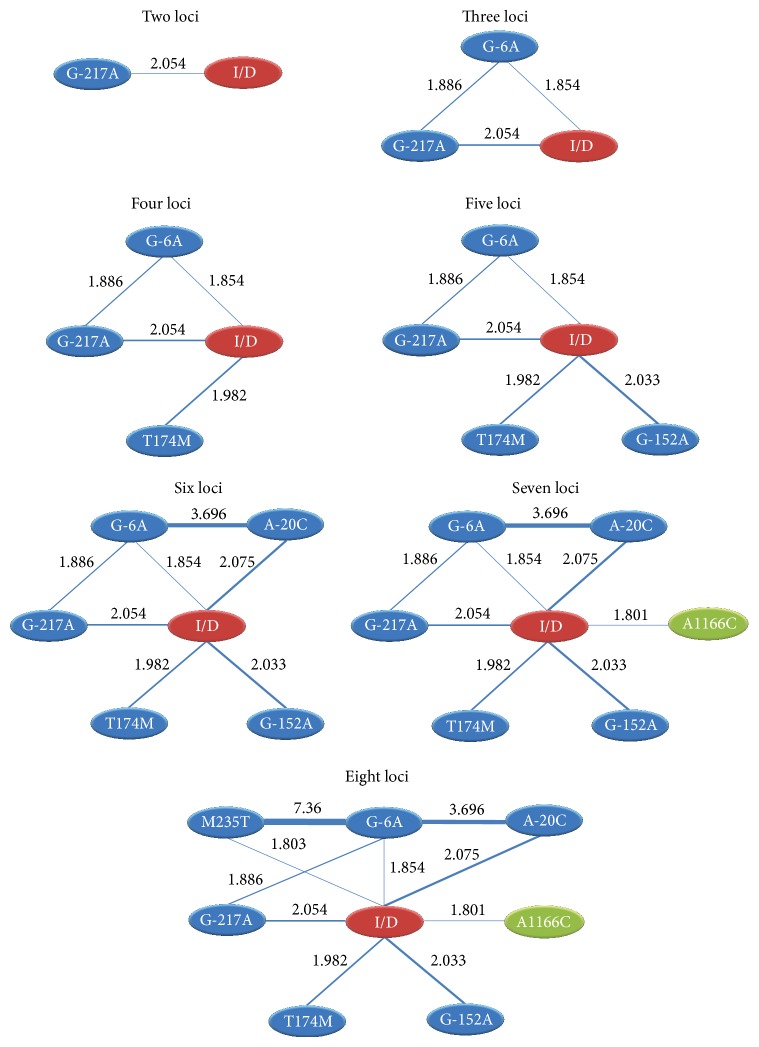
Epistasis networks of the best 2- to 8-locus models for SNP-SNP interaction are associated with hypertension. Significant gene-gene interactions (*P* < 0.05) in these multifoci models are connected by blue lines, and the strength of interaction is labeled with OR values. The thicker and thinner lines represent the higher and lower interactions, respectively.

The SNP-SNP interaction networks (Table 4) have further been validated by the single-SNP-to-single-SNP interaction analyses (Figure 3) for the best 2- to 8-locus models in terms of OR values. SNPs involved in one or more significant interactions are represented as nodes, and the pairs of SNPs with significant interactions are connected by lines. For example, all SNPs are significantly associated with* ACE* I/D, suggesting that* ACE* I/D is mainly associated with hypertension. The G-217A and* ACE* I/D are integrated to the best 2-locus model. The G-6A is further integrated to the best 3-locus model and it is validated that G-6A has positively interacted with G-217A and* ACE* I/D (OR > 1). Similarly, the other newly integrated SNPs in each multiloci model have positively interacted with the previous SNPs in each multiloci model.

Misclassification error is widely used as misclassification performance that aims to correctly estimate the proportions for an incorrect prediction. In MDR, the incorrect prediction error is an internal validation of a measurement that protects against finding chance associations in the sample. The misclassification errors of these multiloci models are much lower than 50%, indicating that the chance associations are significantly reduced. Table 4 also shows that the error rates are gradually reduced from low- to high-order interaction, suggesting that our proposed models are much effective for misclassification of risk of diseases.

In conclusion, hypertension is resulting from the interaction of several genetic risk factors. Analyses of multiple SNP-SNP interactions are complex and remain computational challenges when huge numbers of genetic factors are simultaneously considered. Moreover, the unbalanced data set may have to be analyzed with a bias due to the limitation nature of the MDR approach. In contrast, our proposed algorithm can constitute a nonparametric statistical analysis and provide a model-free and high-order-way measurement for epistasis without the limitation of a balanced data set. Accordingly, a significant outcome can be discovered from the high-order SNP-SNP interaction model amongst an unbalanced data set of many diseases including hypertension. Our results suggest that* AGT*,* ACE*, and* AT*_1_ *R* genes have an overall hypertension susceptibility effect. Among them, SNP I/D of* ACE* has the main association effect to hypertension and it also displays* n*-order interaction effect to SNPs of* AGT* and* AT*_1_ *R* genes although they do not have a mutual effect on each other. The unbalanced function based MDR model can explore the epistasis network of SNPs* AGT*,* ACE*, and* AT*_1_ *R* of RAS genes and identify strongly significant hypertension association. These interaction models and epistasis networks amongst* AGT*,* ACE*, and* AT*_1_ *R* genes may be regarded as potential biomarkers for hypertension susceptibility. Our results also demonstrate that this powerful method has a potential to identify several disease-associated multiloci models as susceptibility biomarkers.

## Supplementary Material

An illustrative example was provided to demonstrate how the unbalanced function based MDR works.

## Figures and Tables

**Table 1:** Characteristics of the study population.

Variables	Entire population (*n* = 443)		*P *values
	HT (*n* = 313)	Non-HT (*n* = 130)	
Gender (M/F)	197/116	98/32	0.008
Age (years)	59.40 ± 11.59	53.55 ± 14.56	<0.001
BH (cm)	161.34 ± 10.53	163.52 ± 11.18	0.092
BW (kg)	65.20 ± 10.59	64.65 ± 14.08	0.720
BMI (m^2^)	25.52 ± 9.89	26.14 ± 27.02	0.820
Smoking (%)	42%	49%	0.235
BP(S) (mmHg)	154.30 ± 14.33	116.64 ± 12.13	<0.001
BP(D) (mmHg)	93.68 ± 10.12	73.34 ± 8.54	<0.001
TG (mg/dl)	146.53 ± 83.87	162.13 ± 96.47	0.209
CHO (mg/dl)	205.77 ± 42.03	192.32 ± 49.18	0.035

BH: body height; BW: body weight; BMI: body mass index; BP(S): systolic blood pressure; BP(D): diastolic blood pressure; TG: triglyceride; CHO: cholesterol.

**Table 2:** Single-locus analysis of eight SNPs for hypertension and nonhypertension groups.

Loci	Genotypes	HT (*n* = 313)	Non-HT (*n* = 130)	*P* values
*AGT* gene				
T174M (rs4762)	CC	243 (77.6%)	106 (81.5%)	
	CT	64 (20.4%)	21 (16.2%)	0.303
	TT	6 (1.9%)	3 (2.3%)	0.874
	C : T	7.2 : 1	8.6 : 1	
M235T (rs699)	CC	220 (70.3%)	92 (70.8%)	
	CT	84 (26.8%)	38 (29.2%)	0.734
	TT	9 (2.9%)	0 (0.0%)	
	C : T	5.1 : 1	5.8 : 1	
G-6A (rs5051)	AA	213 (68.1%)	90 (69.2%)	
	AG	88 (28.1%)	40 (30.8%)	0.749
	GG	12 (3.8%)	0 (0.0%)	
	A : G	4.6 : 1	5.5 : 1	
A-20C (rs5050)	AA	295 (94.2%)	125 (96.2%)	
	AC	15 (4.8%)	3 (2.3%)	0.232
	CC	3 (1.0%)	2 (1.5%)	0.619
	A : C	28.8 : 1	36.1 : 1	
G-152A (rs11568020)	GG	289 (92.3%)	120 (92.3%)	
	GA	21 (6.7%)	10 (7.7%)	0.589
	AA	3 (1.0%)	0 (0.0%)	
	G : A	22.2 : 1	25.0 : 1	
G-217A (rs5049)	GG	228 (72.8%)	102 (78.5%)	
	GA	64 (20.4%)	25 (19.2%)	0.608
	AA	21 (6.7%)	3 (2.3%)	0.057
	G : A	4.9 : 1	7.4 : 1	
*ACE* gene				
I/D (rs4646994)	II	103 (32.9%)	27 (20.8%)	
	ID	146 (46.6%)	67 (51.5%)	**0.031**
	DD	64 (20.4%)	36 (27.7%)	**0.010**
	I : D	1.3 : 1	0.9 : 1	
*AT*_1_ *R* gene				
A1166C (rs5186)	AA	287 (91.7%)	115 (88.5%)	
	AC	25 (8.0%)	14 (10.8%)	0.339
	CC	1 (0.3%)	1 (0.8%)	0.505
	A : C	22.2	15.3 : 1	

**Table 3:** Two-locus SNP-SNP interactions among eight SNPs assessed by unbalanced function based on MDR^∗^.

2 loci	OR values	*P* values	Error rates
*AGT* T174M + *ACE* I/D	1.982 (1.243–3.160)	0.004	0.423
*AGT* M235T + *AGT* G-6A	7.360 (1.734–31.236)	0.007	0.452
*AGT* M235T + *ACE* I/D	1.803 (1.161–2.799)	0.009	0.429
*AGT* G-6A + *AGT* A-20C	3.696 (1.094–12.492)	0.035	0.468
*AGT* G-6A + *AGT *G-217A	1.886 (1.067–3.331)	0.029	0.451
*AGT* G-6A + *ACE* I/D	1.854 (1.187–2.895)	0.007	0.426
*AGT* A-20C + *ACE* I/D	2.075 (1.244–3.461)	0.005	0.428
***AGT* G-217A + *ACE* I/D**	**2.054 (1.310–3.221)**	**0.003**	**0.419**
*AGT* G-152A + *ACE* I/D	2.033 (1.227–3.369)	0.006	0.428
*ACE* I/D + *AT*_1_ *R* A1166C	1.801 (1.104–2.938)	0.018	0.438

^∗^All 2-locus SNP-SNP interactions are identified by the unbalanced function based on MDR method with significant testing accuracy but not best CVC. Bold type represents the best model in 2-locus SNP-SNP interaction models.

**Table 4:** Multiloci analysis of hypertension using unbalanced function based MDR.

Loci number (SNP combination)	Consistency	Error^a^ (%)	OR values (95% CI)	Power	Boot-OR^b^ (95% CI)	*P* ^c^ values
2 loci (G-217A; *ACE* I/D)	4/10	41.9	2.054 (1.31–3.22)	0.901	1.972 (1.26–3.09)	0.003
3 loci (G-6A; G-217A; *ACE* I/D)	4/10	40.3	2.372 (1.51–3.73)	0.986	2.232 (1.42–3.51)	4.99*E* − 04
4 loci (T174M; G-6A; G-217A; *ACE* I/D)	4/10	38.7	2.810 (1.75–4.52)	0.999	2.759 (1.71–4.44)	2.97*E* − 05
5 loci (T174M; G-6A; G-152A; G-217A; *ACE* I/D)	4/10	37.0	3.241 (1.99–5.28)	0.999	3.240 (1.99–5.29)	2.49*E* − 06
6 loci (T174M; G-6A; A-20C; G-152A; G-217A; *ACE* I/D)	6/10	35.3	3.863 (2.36–6.33)	0.999	3.785 (2.31–6.21)	1.34*E* − 07
7 loci (T174M; G-6A; A-20C; G-152A; G-217A; *ACE* I/D; *AT*_1_ *R*)	6/10	33.8	4.510 (2.77–7.34)	0.999	4.050 (2.49–6.58)	1.63*E* − 08
8 loci (T174M; M235T; G-6A; A-20C; G-152A; G-217A; *ACE* I/D; *AT*_1_ *R*)	10/10	32.6	4.628 (2.84–7.54)	0.999	4.483 (2.76–7.29)	1.48*E* − 09

^a^It was determined empirically by permutation testing.^ b^Bootstrapping 1000 samples. ^c^Chi-square test.

## References

[1] Wu S.-J., Chiang F.-T., Jiang J.-R., Hsu K.-L., Chern T.-H., Tseng Y.-Z. (2003). The G—217A variant of the angiotensinogen gene affects basal transcription and is associated with hypertension in a Taiwanese population. *Journal of Hypertension*.

[2] Tsai C. T., Hwang J. J., Lai L. P., Wang Y. C., Lin J. L., Chiang F. T. (2009). Interaction of gender, hypertension, and the angiotensinogen gene haplotypes on the risk of coronary artery disease in a large angiographic cohort. *Atherosclerosis*.

[3] Tsai C.-T., Fallin D., Chiang F.-T. (2003). Angiotensinogen gene haplotype and hypertension: interaction with ACE gene I allele. *Hypertension*.

[4] Ali A., Alghasham A., Ismail H., Dowaidar M., Settin A. (2013). ACE I/D and eNOS E298D gene polymorphisms in Saudi subjects with hypertension. *Journal of the Renin-Angiotensin-Aldosterone System*.

[5] Zhang N., Cui H., Yang L. (2012). Effect of angiotensin II type i receptor A1166C polymorphism on benazepril action in hypertensive patients: a family-based association test study. *Archives of Pharmacal Research*.

[6] Niu W., Qi Y. (2010). Association of the angiotensin II type i receptor gene 1166 A>C polymorphism with hypertension risk: evidence from a meta-analysis of 16474 subjects. *Hypertension Research*.

[7] Mehri S., Mahjoub S., Hammami S. (2012). Renin-Angiotensin system polymorphisms in relation to hypertension status and obesity in a Tunisian population. *Molecular Biology Reports*.

[8] Turgut S., AkIn F., AkcIlar R., Ayada C., Turgut G. (2011). Angiotensin converting enzyme I/D, angiotensinogen M235T and AT1-R A/C1166 gene polymorphisms in patients with acromegaly. *Molecular Biology Reports*.

[9] Saab Y. B., Gard P. R., Overall A. D. J. (2011). The association of hypertension with renin-angiotensin system gene polymorphisms in the Lebanese population. *Journal of the Renin-Angiotensin-Aldosterone System*.

[10] Srivastava K., Sundriyal R., Meena P. C., Bhatia J., Narang R., Saluja D. (2012). Association of angiotensin converting enzyme (insertion/deletion) gene polymorphism with essential hypertension in Northern Indian subjects. *Genetic Testing and Molecular Biomarkers*.

[11] Kaur R., Das R., Ahluwalia J., Kumar R. M., Talwar K. K. (2012). Synergistic effect of angiotensin II type-1 receptor 1166A/C with angiotensin-converting enzyme polymorphism on risk of acute myocardial infarction in north Indians. *Journal of the Renin-Angiotensin-Aldosterone System*.

[12] Tsai C.-T., Hwang J.-J., Ritchie M. D. (2007). Renin-angiotensin system gene polymorphisms and coronary artery disease in a large angiographic cohort: detection of high order gene-gene interaction. *Atherosclerosis*.

[13] Montasser M. E., Gu D., Chen J. (2011). Interactions of genetic variants with physical activity are associated with blood pressure in Chinese: the GenSalt study. *American Journal of Hypertension*.

[14] Meyers K. J., Chu J., Mosley T. H., Kardia S. L. R. (2010). SNP-SNP interactions dominate the genetic architecture of candidate genes associated with left ventricular mass in African-Americans of the GENOA study. *BMC Medical Genetics*.

[15] Wu S.-J., Chuang L.-Y., Lin Y.-D. (2013). Particle swarm optimization algorithm for analyzing SNP-SNP interaction of renin-angiotensin system genes against hypertension. *Molecular Biology Reports*.

[16] Yang C. H., Chuang L. Y., Cheng Y. H. (2012). Single nucleotide polymorphism barcoding to evaluate oral cancer risk using odds ratio-based genetic algorithms. *Kaohsiung Journal of Medical Sciences*.

[17] Van steen K. (2012). Travelling the world of gene-gene interactions. *Briefings in Bioinformatics*.

[18] Chuang L.-Y., Lin Y.-D., Chang H.-W., Yang C.-H. (2012). An improved PSO algorithm for generating protective SNP barcodes in breast cancer. *PLoS ONE*.

[19] Moore J. H., Asselbergs F. W., Williams S. M. (2010). Bioinformatics challenges for genome-wide association studies. *Bioinformatics*.

[20] Chang H.-W., Yang C.-H., Ho C.-H., Wen C.-H., Chuang L.-Y. (2009). Generating SNP barcode to evaluate SNP-SNP interaction of disease by particle swarm optimization. *Computational Biology and Chemistry*.

[21] Song Y. S., Wang F., Slatkin M. (2010). General epistatic models of the risk of complex diseases. *Genetics*.

[22] Chen S.-H., Sun J., Dimitrov L. (2008). A support vector machine approach for detecting gene-gene interaction. *Genetic Epidemiology*.

[23] Ritchie M. D., Hahn L. W., Roodi N. (2001). Multifactor-dimensionality reduction reveals high-order interactions among estrogen-metabolism genes in sporadic breast cancer. *The American Journal of Human Genetics*.

[24] Hahn L. W., Ritchie M. D., Moore J. H. (2003). Multifactor dimensionality reduction software for detecting gene-gene and gene-environment interactions. *Bioinformatics*.

[25] Yang C. H., Lin Y. D., Chuang L. Y., Chen J. B., Chang H. W. (2013). MDR-ER: Balancing functions for adjusting the ratio in risk classes and classification errors for imbalanced cases and controls using multifactor-dimensionality reduction. *PLoS ONE*.

[26] Velez D. R., White B. C., Motsinger A. A. (2007). A balanced accuracy function for epistasis modeling in imbalanced datasets using multifactor dimensionality reduction. *Genetic Epidemiology*.

[27] Wu S.-J., Chiang F.-T., Chen W. J. (2004). Three single-nucleotide polymorphisms of the angiotensinogen gene and susceptibility to hypertension: single locus genotype vs. haplotype analysis. *Physiological Genomics*.

[28] Faul F., Erdfelder E., Lang A.-G., Buchner A. (2007). G^∗^power 3: a flexible statistical power analysis program for the social, behavioral, and biomedical sciences. *Behavior Research Methods*.

[29] Erdfelder E., FAul F., Buchner A., Lang A.-G. (2009). Statistical power analyses using G∗Power 3.1: tests for correlation and regression analyses. *Behavior Research Methods*.

[30] Sallinen R., Kaunisto M. A., Forsblom C. (2010). Association of the SLC22A1, SLC22A2, and SLC22A3 genes encoding organic cation transporters with diabetic nephropathy and hypertension. *Annals of Medicine*.

[31] Xu J., Ji L.-D., Zhang L.-N. (2013). Lack of association between STK39 and hypertension in the Chinese population. *Journal of Human Hypertension*.

[32] Polimanti R., Piacentini S., Lazzarin N., Re M. A., Manfellotto D., Fuciarelli M. (2012). Lack of association between essential hypertension and *GSTO1* uncommon genetic variants in Italian patients. *Genetic Testing and Molecular Biomarkers*.

[33] Su G., Christensen O. F., Ostersen T., Henryon M., Lund M. S. (2012). Estimating additive and non-additive genetic variances and predicting genetic merits using genome-wide dense single nucleotide polymorphism markers. *PLoS ONE*.

