# Supplementary material for: High Order Gene-Gene Interactions in Eight Single Nucleotide Polymorphisms of Renin-Angiotensin System Genes for Hypertension Association Study
Source: Biomed Res Int. 2015 Apr 19;2015:454091. doi: 10.1155/2015/454091 (PMC4417588; doi:10.1155/2015/454091)
Supplement: Supplementary file 1 — An illustrative example was provided to demonstrate how the unbalanced function based MDR works. [file 454091.f1.docx]

ONLINE SUPPLEMENT

**Detection of high order gene-gene interactions among three renin-angiotensin system (RAS) gene polymorphisms in hypertension**

Cheng-Hong Yang^1^, Yu-Da Lin^1^, Shyh-Jong Wu^2*^, Li-Yeh Chuang^3*^, and Hsueh-Wei Chang^4,5,6,7*^

^1^ Department of Electronic Engineering, National Kaohsiung University of Applied Sciences, Kaohsiung, Taiwan.

^2^ Department of Medical Laboratory Science and Biotechnology, Kaohsiung Medical University, Kaohsiung, Taiwan.

^3^ Department of Chemical Engineering & Institute of Biotechnology and Chemical Engineering, I-Shou University, Kaohsiung, Taiwan.

^4^ Cancer Center, Translational Research Center, Kaohsiung Medical University Hospital; Kaohsiung Medical University, Kaohsiung, Taiwan.

^5^ Institute of Medical Science and Technology, National Sun Yat-sen University, Kaohsiung, Taiwan.

^6^ Research Center of Environmental Medicine, Kaohsiung Medical University, Kaohsiung, Taiwan.

^7^ Department of Biomedical Science and Environmental Biology, Kaohsiung Medical University, Kaohsiung, Taiwan.

**Supplemental method example**

Here we provide an illustrative example to show how the unbalanced function based MDR works in supplementary file. The symbols AA, Aa, and aa represent the three genotypes of locus_1_, and the symbols BB, Bb, and bb represent the three genotypes of locus_2_. The steps of the unbalanced function based MDR approach are shown as follows.

**Step 1)** Divide the data set into 10 subsets for cross-validation (CV).

**Step 2)** Let the *i*^th^ data set as the testing data and others are the training data.

**Step 3)** Calculate the total number of cases and the total number of controls within each multi-factor class.

For example, the sample distributions in the two loci are calculated as following:

*D*_locus1,_ _locus2_ = [*x*_AA,BB_, *x*_AA,Bb_, *x*_AA,bb_, *x*_Aa,BB_, *x*_Aa,Bb_, *x*_Aa,bb_, *x*_aa,BB_, *x*_aa,Bb_, *x*_aa,bb_]

= [(11, 33)_AA,BB_, (19, 54)_AA,Bb_, (57, 280)_AA,bb_,

(25, 45)_Aa,BB_, (16, 43)_Aa,Bb_, (17, 55)_Aa,bb_,

(13, 27)_aa,BB_, (10, 33)_aa,Bb_, (37, 87)_aa,bb_].

**Step 4)** Evaluate the ratio between cases and controls.

For example

(11, 33)_AA,BB_ = (11×657)/(33×205) = 1.068

(19, 54)_AA,Bb_ = (19×657)/(87×205) = 1.128

(57, 280)_AA,bb_ = (57×657)/(280×205) = 0.652

(25, 45)_Aa,BB_ = (25×657)/(45×205) = 1.780

(16, 43)_Aa,Bb_ = (16×657)/(43×205) = 1.193

(17, 55)_Aa,bb_ = (17×657)/(55×205) = 0.991

(13, 27)_aa,BB_ = (13×657)/(27×205) = 1.543

(10, 33)_aa,Bb_ = (10×657)/(33×205) = 0.971

(37, 87)_aa,bb_ = (37×657)/(87×205) = 1.363.

**Step 5)** Determine the high/low risk in each multifactor class. Label ‘H’ if the ratio ≥ particular threshold; otherwise it is labeled ‘L’.

When the ratio value is bigger than a threshold of *T* = 1, the cell is classified as high-risk; otherwise, the cell is classified as low-risk. The generated labels are shown as following.

∵ (11, 33)_AA,BB_ ≥ *T* = 1 ∴ L_AA,BB_ = ‘H’

∵ (19, 54)_AA,Bb_ ≥ *T* = 1 ∴ L_AA,Bb_ = ‘H’

∵ (57, 280)_AA,bb_ < *T* = 1 ∴ L_AA,bb_ = ‘L’

∵ (25, 45)_Aa,BB_ ≥ *T* = 1 ∴ L_Aa,BB_ = ‘H’

∵ (16, 43)_Aa,Bb_ ≥ *T* = 1 ∴ L_Aa,Bb_ = ‘H’

∵ (17, 55)_Aa,bb_ < *T* = 1 ∴ L_Aa,bb_ = ‘L’

∵ (13, 27)_aa,BB_ ≥ *T* = 1 ∴ L_aa,BB_ = ‘H’

∵ (10, 33)_aa,Bb_ < *T* = 1 ∴ L_aa,Bb_ = ‘L’

∵ (37, 87)_aa,bb_ ≥ *T* = 1 ∴ L_aa,bb_ = ‘H’.

**Step 6)** Compute the four frequencies (TP, FP, TN, and FN) in a 2-way contingency table.

The step sums the total number of high- and low-risk among the cases and controls.

*H* ={L_AA,BB_, L_AA,Bb_, L_Aa,BB_, L_Aa,Bb_, L_aa,BB_, L_aa,bb_ | L∈‘H’}

=> H_case, control_ = {(11, 33)_AA,BB_, (19, 54)_AA,Bb_, (25, 45)_Aa,BB_, (16, 43)_Aa,Bb_, (13, 27)_aa,BB_, (37, 87)_aa,bb_}

=> H_case_ = 11+19+25+16+13+37 = 121; H_control_ = 33+54+45+43+27+87 = 289

=> TP = 121; FP = 289.

*L* ={L_AA,bb_, L_Aa,bb_, L_aa,Bb_ | L∈‘L’}

=> L_case, control_ = {(57, 280)_AA,bb_, (17, 55)_Aa,bb_, (10, 33)_aa,Bb_}

=> L_case_ = 57+17+10 = 84; L_control_ = 280+55+33 = 368

=> FN = 84; TN = 368.

**Step 7)** Evaluate the classification error.

The error rates of MDR-E and MDR-ER are computed by equation 3.

*$\begin{matrix} Error\_rate_{\text{Locus1,Locus2}}=0.5\times\left(\frac{FN}{TP+FN}+\frac{FP}{FP+TN}\right) \\ \text{ }=0.5\times\left(\frac{84}{121+84}+\frac{289}{289+368}\right) \\ \text{ }=0.425 \end{matrix}$*

**Step 8)** Repeat for each combination and record the error_rate into a set *E*.

The error_rate is record into a set

*E_i_* = {*e* _locus1,_ _locus2_, *e* _locus1,_ _locus3_, …, *e* _locus_*_n-1_*_,_ _locus_*_n_*}.

where *n* is the total number of SNPs.

If all combinations are evaluated, then go to Step 9; otherwise, go to Step 3.

**Step 9)** Select best model according classification error and record into cross-validation consistency.

The best model is record into a set cross-validation consistency

*CVC* = {*c*_1_, *c*_2_, …, *c*_10_ | c*_i_*∈MIN(*E_i_*)}

**Step 10)** Repeat for each cross-validation interval.

If all cross-validation intervals are evaluated, then go to Step 11; otherwise, go to Step 2.

**Step 11)** Select best model according *CVC*.

The best model is the most events appeared in *CVC*.
